# Topical-Ozonized Olive Oil – A Boon for Post-Extraction Cases: A Randomized Controlled Trial

**DOI:** 10.7759/cureus.34478

**Published:** 2023-01-31

**Authors:** Ayush Satapathy, Abhishek Balani, Vinay Kharsan, Abhishek Karan, Heena Mazhar, Arunima Awasthy

**Affiliations:** 1 Department of Oral and Maxillofacial Surgery, New Horizon Dental College and Research Institute, Bilaspur, IND

**Keywords:** ozonized, olive oil, analgesics, antibiotics, oral surgery, pain, exodontia, topical ozone

## Abstract

Background

Post-surgical therapy in exodontia patients has historically been largely centered on pain and infection prevention. Healing of the extraction wound has rarely received any importance during regular dental extractions, despite being an inherent element of the process of tooth extraction itself. This study aimed to analyze the analgesic and antibacterial efficacy of topical-ozonized olive oil compared to regular drugs administered post-operatively to patients who have undergone tooth extraction as well as evaluate the healing effects of the former on the extraction site.

Methodology

A total of 200 patients in need of exodontia were randomly divided into two groups, with group A (case group) receiving ozonized olive oil as a topical application for three days and group B (control group) receiving standard post-operative treatment (antibiotics and analgesics). On day five, patients in both groups were assessed for wound healing using the Landry, Turnbull, and Howley Index and for pain using the visual analog scale (VAS).

Results

On days two and three, the P-value for differences in pain (VAS score) between the two groups was 0.409, but on day five, it was 0.180. According to the Landry, Turnbull, and Howley index, the P-value for differences in wound healing between the groups on day five was 0.025. When comparing the two groups, there was no discernible difference in the amount of discomfort perceived after surgery. While both groups saw improvement in wound healing and pain, the case group coped better than the control group in terms of wound healing.

Conclusions

This study demonstrated that ozonized olive oil may be used as a safe and effective alternative to conventional painkillers and antibiotics and can speed up wound healing after exodontia.

## Introduction

Since the dawn of time, ozone has been in the atmosphere and shielded humans on earth from the sun’s harmful ultraviolet radiation. The word ozone comes from the Greek word ‘Ozein’ meaning “odor” based on its characteristic pungent odor [1]. Ozone is a potent oxidant since it is a very unstable allotrope of oxygen. In 1870, Dr. C. Lender was the first to utilize ozone for medicinal purposes; he used ozone to sterilize blood samples in a laboratory. Ozone has antimicrobial properties against gram-positive and gram-negative bacteria, viruses, and fungi. Its cellular biological characteristics in terms of biocompatibility make it appropriate for oral application: ozonized water has the ability to accelerate the epithelial healing of the oral cavity, especially during the first two post-operative days. Ozonized water (0.5-4 mg/ml) is effective in killing gram-positive microorganisms present in the dental biofilm and inhibiting the accumulation of experimental plagues in vitro [2]. Ozone has been used in dentistry for a wide range of purposes, including wound healing, dental caries, oral lichen planus, gingivitis and periodontitis, halitosis, osteonecrosis of the jaw, post-surgical pain, plaque and biofilms, root canals, dentin hypersensitivity, temporomandibular joint disorders, and teeth whitening [3]. Nowadays, ozone is routinely used in oral and maxillofacial surgery. The use of ozone in the form of ozonized water as an irrigant during third molar extractions, as well as the effectiveness of ozone gas and ozonized olive oil in healing the extraction wound following the procedure, has been shown in studies to improve infection, inflammation, and pain control, as well as speed up and improve wound healing [2-5]. With the post-operative use of ozone after third molar surgeries, it has been found that patients required fewer analgesics. Therefore, we conducted a study to evaluate the analgesic and antimicrobial efficiency and wound healing properties of topical ozonized olive oil as a potent alternative to conventional medications (antibiotics and analgesics) for post-operative care of standard interalveolar extractions.

## Materials and methods

**Figure 1:**
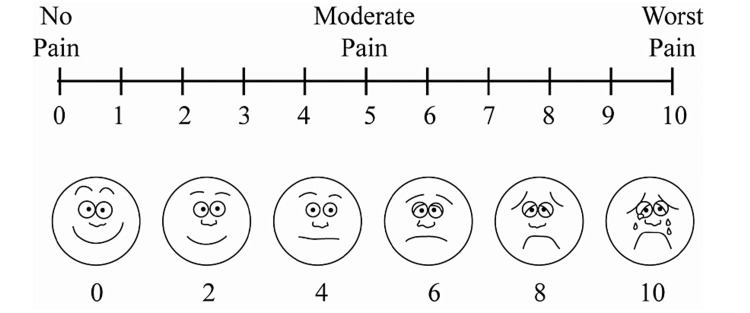
Visual analog scale.

Patients between the ages of 18 and 50 who sought care at the outpatient clinic of the department of oral and maxillofacial surgery at New Horizon Dental College and Research Institute in Bilaspur, Chhattisgarh, between April 2021 and March 2022 were enrolled in this randomized controlled clinical trial. The sample size of 200 was determined using the Cochran formula. The research began after approval from the institution’s dean, scientific advisory board, and ethical review board (ref no: NHDCRI/2020/11). Before being enrolled in the experiment, all patients provided written, signed informed consent. The exodontia patients in the sample were randomly divided into groups A and B. Patients in Group A (case group) applied ozonized olive oil (100% pure ozone) on their wound once daily for three days following the surgery, while those in Group B (control group) received the standard post-operative care of antibiotics (amoxicillin 500 mg + clavulanic acid 125 mg) twice daily for seven days and analgesics (aceclofenac 100 mg + paracetamol 325 mg) twice daily for three days. Those in Group B who experienced gastrointestinal distress after taking analgesics were given a proton pump inhibitor (pantoprazole 40 mg) once a day, 30 minutes before food. Before the surgery, as well as on days two, three, and five, patients in both groups were asked to rate their pain using a visual analog scale (VAS) to estimate their recovery progress. All patients were pre-operatively evaluated for their tooth region pain tolerance using a VAS. The VAS consists of a 10 cm line, with two endpoints representing 0 (no pain) and 10 (the highest-possible pain) (Figure 1).

On the fifth day after the extraction, the wound healing of patients in both groups was evaluated using the Landry, Turnbull, and Howley index, in which the combination of presence or absence of five clinical criteria determines the healing index with a range of 1 (very poor) to 5 (excellent). Tooth extractions that took more than 15 minutes were excluded from the study because the researchers feared that the longer operating duration might affect the reliability of the results. As the healing parameters were mostly subjective, they were evaluated by an observer who was unaware of the randomization or therapies.

For randomization, patients who presented for treatment with odd outpatient department (OPD) numbers were assigned to the case group by the department nurse and those with even OPD numbers were assigned to the control group. One author, who was blinded to this randomization, assessed the patients in each group post-operatively.

The inclusion criteria include intra-alveolar simple extractions; patients requiring tooth extraction due to periodontal or decay etiology; extraction of single-rooted and multi-rooted teeth; and patients aged 20-45 years. Exclusion criteria include patients with sinus opening and severe infection, pregnancy, systemic disease, patients receiving antibiotic therapy, a history of antibiotic therapy, chemotherapy, myocardial infarction, ozone allergy, alcohol abuse, hyperthyroidism, and severe anemia, and those extractions complicated intra-operatively, leading to a more invasive and longer procedure.

Methodology

**Figure 2:**
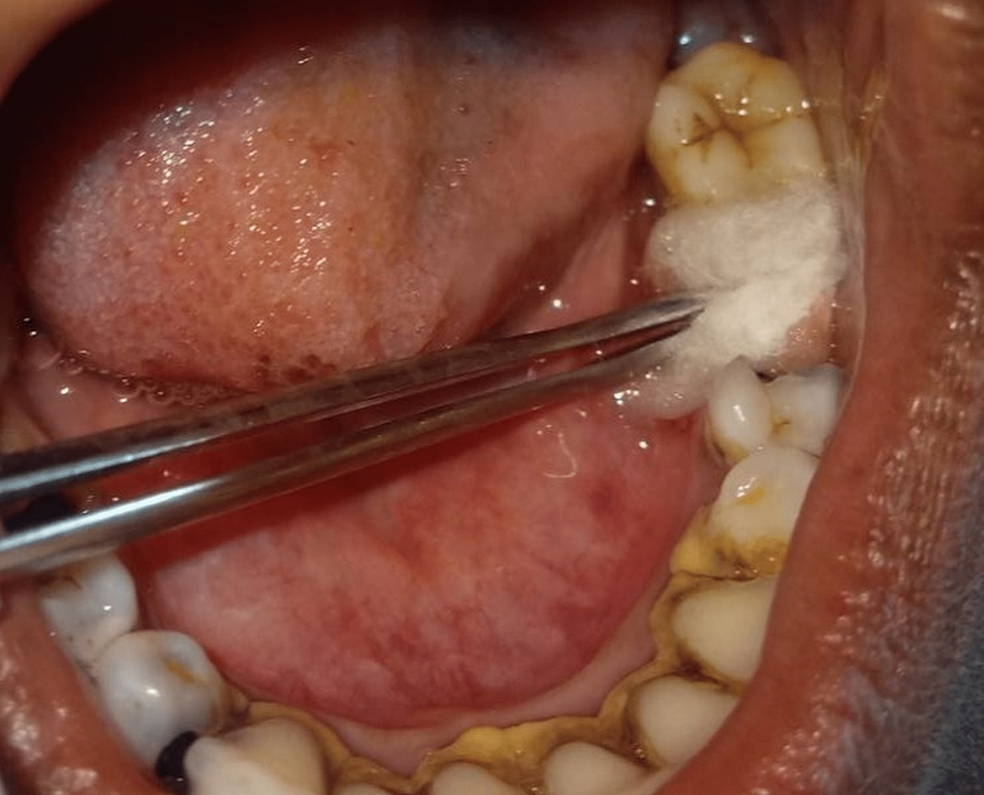
Application of ozone oil in the extracted site

**Figure 3:**
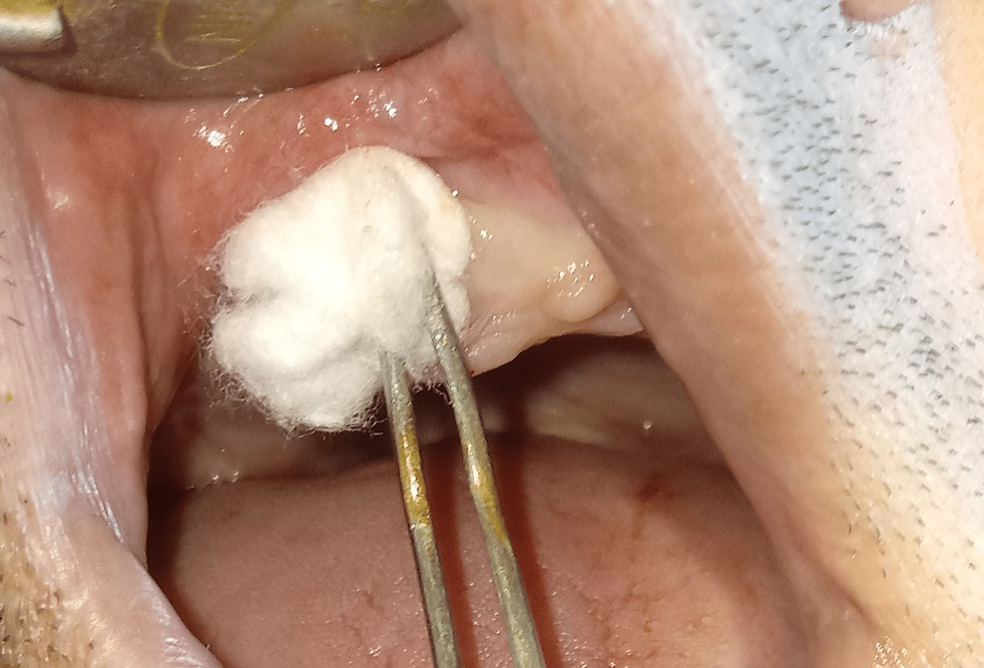
Application of ozone oil in the extracted site

Patients in the case group had intra-alveolar simple teeth extracted while under local anesthesia (2% lidocaine with 1:80,000 adrenaline). Afterward, cotton soaked in ozonized olive oil was applied to the extraction site. (Figure 2 and Figure 3).

 

After two minutes of holding cotton onto the extraction site with a tweezer, the cotton was removed and the patient was given a pressure pack and sent away with post-operative instructions for tooth extraction as well as a stern instruction to not take any medication without visiting the operator. Case group patients were called again for the next two days for further application of the oil and had their pain levels assessed by an independent observer using the VAS, where, using a ruler, the score was determined based on the distance (mm) between the “no pain” anchor and the patient’s mark. A higher VAS score indicates greater pain intensity. Patients in the control group were provided with a pressure pack, post-operative instructions, standard analgesics (aceclofenac 100 mg and paracetamol 325 mg) twice daily for three days and antibiotics (amoxicillin 500 mg and clavulanic acid 125 mg) twice daily for a period of seven days. Patients in the control group who reported experiencing stomach irritation after taking analgesics were given a proton-pump inhibitor (pantoprazole 40 mg) once a day, 30 minutes before food. For two consecutive days, the same blinded observer who evaluated the pain of the case group also evaluated the pain of the control group using a VAS. 

**Figure 4:**
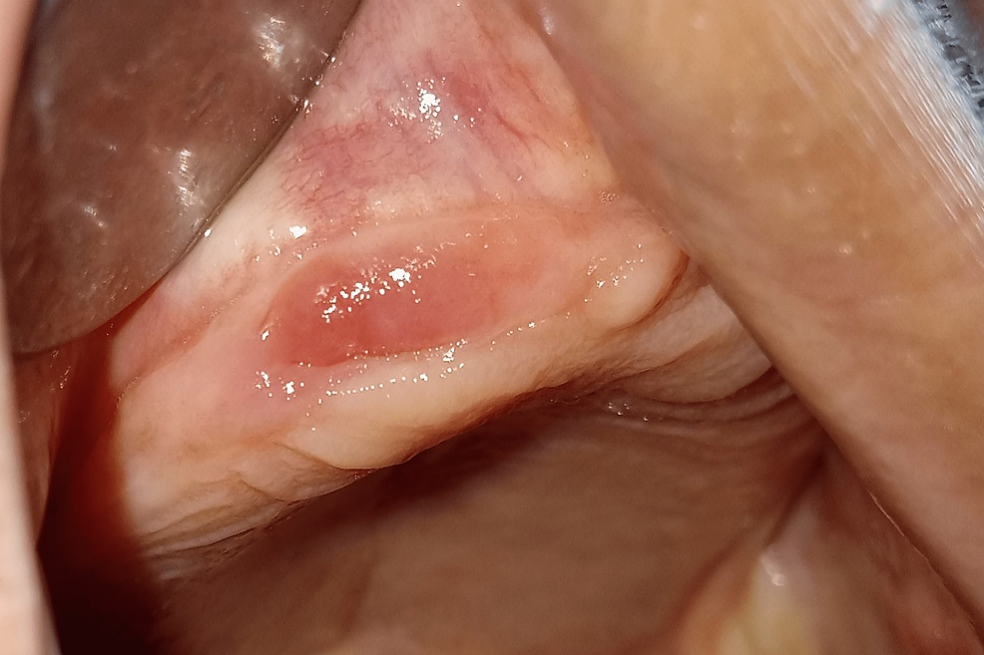
Healing after extraction

The statistical analysis using an independent t-test was performed in the SPSS V.24 software (Statistical Package for the Social Sciences). The p-value of 0.05 was considered statistically significant.

## Results

A total of 294 individuals were initially evaluated for the research using a basic randomized sample procedure; however, only 237 were ultimately included since 33 patients could not meet the inclusion criteria and 14 patients declined to participate. Group A had 128 patients, whereas Group B had 119 patients. Group A had a loss of 17 patients due to non-fulfilment of inclusion criteria or withdrawal, whereas group B had a loss of 19 patients. Finally, 100 patients in each group were provided with the therapy they were supposed to, and the main result could be assessed.

In the sample, there were more male patients in both case and control groups (113 and 107, respectively) as compared to female patients (87 and 93 respectively); most patients were in the age range of 31-45 years in the case group and 18-30 years in the control group. Maximum mandibular posteriors were indicated for extraction, of which 91 patients were in the case group and 89 patients were in the control group. Maximum teeth indicated for extraction were from the mandibular right side in both case and control groups (61 and 59, respectively). Patients undergoing extraction due to pulpitis showed comparatively poor pain tolerance and healing than patients undergoing extraction due to any other etiology in both case and control groups. No difficulty index was considered for assessing the extractions, as none of the cases included was surgical extractions.

The mean comparison between preoperative pain using the VAS score showed a P-value of 0.442, a T-value of 0.776, and a mean difference of 0.48. The mean comparison between post-operative pain using the VAS score on day two showed a P-value of 0.409, a T-value of 0.832, and a mean difference of 0.32. The mean comparison between post-operative pain using the VAS score on day three showed a P-value of 0.180, a T-value of 1.361, and a mean difference of 0.48. The mean comparison between post-operative pain using the VAS score on day five showed a P-value of 0.070, a T-value of 1.857, and a mean difference of 0.36 (Table 1).

**Table 1:** Comparison of pre-operative and postoperative VAS scores between the case and control groups. SD: standard deviation; VAS: visual analog scale

Days	Groups	Mean	SD	Mean difference	T-value	P-value
Preoperative	Case	5.6	2.78	0.48	0.776	0.442
	Control	6.08	1.35			
Day two	Case	2.24	1.5	0.32	0.832	0.409
	Control	2.56	1.19			
Day three	Case	1.12	1.23	0.48	1.361	0.18
	Control	1.6	1.25			
Day five	Case	0.24	0.43	0.36	1.857	0.07
	Control	0.6	0.86			

The mean comparison between post-operative wound healing on day seven using Landry, Turnbull, and Howley index between the case and control groups showed a P-value of 0.025, a T-value of 2.313, and a mean difference of 0.48 (Table 2).

**Table 2:** Comparison of wound healing between the case and control groups. * Statistically significant

Groups	Mean	SD	Mean difference	T value	P value
Case	4.40	0.70	0.48	2.313	0.025*
Control	3.92	0.75			

## Discussion

In many studies, the management of primary Sjögren syndrome due to tooth extraction was examined in relation to alternative natural materials. For instance, a randomized, split-mouth controlled research on 33 participants examined the impact of intrasocket Manuka honey administration on post-operative pain. Study participants who consumed Manuka honey had lower VAS scores on the first and second days [6]. According to one study, using Arnica Montana supplements significantly reduced post-operative edema, bruising, and trismus without significantly increasing toxicity [7]. Due to its prevalence as an invasive surgical operation in dental offices, exodontia has a well-deserved reputation for being one of the most taxing procedures for patient psyches. The psychological state of the patient and pain associated with the procedure are very likely to cause the development of analgesic dependency in the patient, which brings along with it the vast array of adverse effects associated with the prolonged use of analgesics [8-16]. Exodontia is ideally performed under the utmost sterile conditions using properly sterilized equipment; thus, the requirement of antibiotics in routine exodontia can be considered irrelevant if the procedure is conducted properly. However, in order to prevent any post-operative complications due to a lack of patient compliance or deleterious habits of the patient, both the patient and the practitioner prefer to opt for a post-operative antibiotic regimen. Although post-operative antibiotics offer the advantage of infection control, it also sometimes results in adverse effects such as diarrhea, nausea, vomiting, and allergic reactions [17]. Nowadays, proper post-operative healing of the socket and implant-suitable alveolar ridge remodeling with adequate soft and hard tissue volume are essential considerations during exodontia in order to facilitate optimum prosthetic rehabilitation for the patient [15].

Being an unstable compound, ozone stimulates the production of oxidizing free radicals which selectively damage the cell wall and plasma membrane of microorganisms by hampering their enzymatic metabolism, thereby acting as a potent antimicrobial agent [1,11,18,19,20,21]. The anti-inflammatory action of ozone is attributed to its ability to promote the production of biologically active compounds such as leukotrienes, prostaglandins, and interleukins, which are known for their anti-inflammatory and analgesic actions. Neovascularization in inflamed tissues and promotion of oxygen supply to hypoxic tissues by a free radical system of ozone optimizes the healing of both hard and soft tissues, which are essential for proper prosthetic rehabilitation of the edentulous site [11].

The purpose of this study was to evaluate the efficacy of ozone in oral surgical practice and to provide a safe alternative to conventional analgesics and antibiotics, both of which have undesirable side effects. A total of 200 patients between the ages of 20 and 45 comprised the study sample. Participants were subsequently separated into a case group (group A) and a control group (group B), with the former receiving ozonized olive oil for three days after surgery and the latter receiving standard post-operative treatment with antibiotics and analgesics. Specifically, the aim of this research was to evaluate the efficacy of applying ozonized olive oil topically to the extraction socket for three consecutive days following the extraction as a viable alternative to the standard treatment with analgesics and antibiotics, as well as a powerful healing agent. All the patients with underlying systemic disorders were excluded from the study to eliminate any influence of the systemic condition of the patient on the treatment outcomes. The study protocol included the topical application of ozonized olive oil on the extraction site for two minutes once a day post-operatively for three days using cotton and a tweezer. The pain assessment of the patients was done using a VAS. The P-value of the VAS score of the two groups for days two, three, and five was 0.409, 0.180, and 0.070, respectively. The wound healing was estimated using the Landry, Turnbull, and Howley index, and the P-value for the wound healing of the two groups on day five was 0.025. The P-value for preoperative VAS scale comparison was 0.442, and the healing index comparison for easy and difficult extractions was 0.284.

The results of the study clearly prove the effectiveness of ozonized olive oil over routine analgesics and antibiotics. This study’s findings on ozone’s role in routine interalveolar extractions are consistent with those of other research on a similar topic. The effectiveness of gaseous ozone in surgical third molar extraction was evaluated by Ahmedi et al. [1], and the authors found that the use of ozone decreased the occurrence of dry sockets (albeit not significantly, P = 0.20) and the need for analgesics. Ramos et al. [2] studied the influence of ozonized water on post-operative trismus, edema, and pain during surgery of the impacted third molar and confirmed its efficacy. Kumar et al. [6] examined the effect of ozonized olive oil on the management of oral lesions and conditions and found accelerated regression of lesions and improvement in signs and symptoms with no toxicity or adverse effects. Bahl et al. [22] demonstrated in their prospective study that patients who applied ozonized olive oil post-operatively at the extraction site, without antibiotic and analgesic therapy on an SOS basis showed less post-operative pain and swelling, better wound healing, and lesser analgesic dependency. 

Rusdy et al. [23] asked their patients to bite on a tampon soaked in ozonated water following tooth extraction for 30 minutes and then checked the inflammation after 30 minutes and again after one week using the Gingival Score Index. They came to the conclusion that ozone water might minimize inflammation of the tooth socket and speed up the wound healing process. Despite the significantly positive results of this research, a few limitations were found. The three or more days of recall for subsequent applications were tedious for the patient and the operator. Thus, a better dispensing medium for self-administration by the patient is required. Some patients complained of mild aggravation of pain on subsequent mornings between the subsequent applications, which might be attributed to the exhaustion of the action of ozone; this can be corrected by increasing the frequency of application to two times a day from once a day. As the application is topical, pain during the action of muscles of mastication and other deep structure cannot be avoided in the cases of third molar or traumatic extractions [24,25]. A medium that can facilitate the gradual prolonged release of ozone will be very helpful in managing the pain for a longer time period and can be placed into the socket of simple intralveolar extractions and surgically extracted third molars to estimate the horizons of the efficacy of ozone in pain management. Compromise in sterilization protocol leading to the need for antibiotics post-operatively is completely unacceptable. Proper post-operative instructions and counselling should be given to patients in order to avoid unnecessary and prolonged analgesic and antibiotic intake. The application of ozonized olive oil is unquestionably a reasonable alternative to common analgesics and antibiotics, which may be safe and effective.

## Conclusions

Ozonized olive oil is as effective as conventional analgesics and antibiotics for the post-operative management of pain and inflammation. This study clearly demonstrated that the administration of topical ozone to wounds in the cases of exodontia improves and accelerates the healing process, establishing its superiority over standard therapies. The development of better dispensing methods suitable for self-administration by the patient and prolonged gradual action of ozone at the target site will be vital in making topical ozonized olive oil a part of the routine armamentarium for dental extraction.

## References

[1] Ahmedi J, Ahmedi E, Sejfija O, Agani Z, Hamiti V (2016). Efficiency of gaseous ozone in reducing the development of dry socket following surgical third molar extraction. Eur J Dent.

[2] Glória JC, Douglas-de-Oliveira DW, E Silva LD, Falci SG, Dos Santos CR (2020). Influence of ozonized water on pain, oedema, and trismus during impacted third molar surgery: a randomized, triple blind clinical trial. BMC Oral Health.

[3] Suh Y, Patel S, Kaitlyn R, Gandhi J, Joshi G, Smith NL, Khan SA (2019). Clinical utility of ozone therapy in dental and oral medicine. Med Gas Res.

[4] Sandhu RK (2021). Ozone in Dentistry-A review. Adv Med Dent Sci Research.

[5] Sivalingam VP, Panneerselvam E, Raja KV, Gopi G (2017). Does topical ozone therapy improve patient comfort after surgical removal of impacted mandibular third molar? A randomized controlled trial. J Oral Maxillofac Surg.

[6] Al-Khanati NM, Al-Moudallal Y (2019). Effect of Intrasocket Application of Manuka Honey on Postsurgical Pain of Impacted Mandibular Third Molars Surgery: Split-Mouth Randomized Controlled Trial.. J Maxillofac Oral Surg.

[7] Mawardi H, Ghazalh S, Shehatah A (2020). Systemic use of Arnica montana for the reduction of postsurgical sequels following extraction of impacted mandibular 3(rd) molars: a pilot study. Evid Based Complement Alternat Med.

[8] Kumar T, Arora N, Puri G, Aravinda K, Dixit A, Jatti D (2016). Efficacy of ozonized olive oil in the management of oral lesions and conditions: A clinical trial. Contemp Clin Dent.

[9] Steier L (2004). Ozone: The Revolution in Dentistry. Quintessence Publishing.

[10] Loncar B, Mravak Stipetic M, Matosevic D, Tarle Z (2009). Ozone application in dentistry. Arch Med Res.

[11] Makkar S, Makkar M (2011). Ozone-treating dental infections. Indian J Stomatol.

[12] Seidler V, Linetskiy I, Hubálková H, Stanková H, Smucler R, Mazánek J (2008). Ozone and its usage in general medicine and dentistry. A review article. Prague Med Rep.

[13] Huth KC, Jakob FM, Saugel B (2006). Effect of ozone on oral cells compared with established antimicrobials. Eur J Oral Sci.

[14] Nogales CG, Ferrari PH, Kantorovich EO, Lage-Marques JL (2008). Ozone therapy in medicine and dentistry. J Contemp Dent Pract.

[15] Azarpazhooh A, Limeback H (2008). The application of ozone in dentistry: a systematic review of literature. J Dent.

[16] Bocci VA (2006). Scientific and medical aspects of ozone therapy. State of the art. Arch Med Res.

[17] Sechi LA, Lezcano I, Nunez N (2001). Antibacterial activity of ozonized sunflower oil (Oleozon). J Appl Microbiol.

[18] Rodrigues KL, Cardoso CC, Caputo LR, Carvalho JC, Fiorini JE, Schneedorf JM (2004). Cicatrizing and antimicrobial properties of an ozonised oil from sunflower seeds. Inflammopharmacology.

[19] Said O, Elander J, Maratos FA (2019). An international study of analgesic dependence among people with pain in the general population. Subst Use Misuse.

[20] Pérez-Amate B, Figueiredo R, Cortés-Peral S, Sánchez-Torres A, Valmaseda-Castellón E (2021). Patient perception about the need for antibiotics after tooth extractions: a cross-sectional study. J Clin Exp Dent.

[21] Liaqat S, Tariq S, Hayat I (2022). Therapeutic effects and uses of ozone in dentistry: A systematic review. Ozone Sci Eng.

[22] Bahl D, Samuel S, Charyulu RN, Dole S (2021). Use of topical ozone therapy for wound healing after transalveolar extractions: a miracle alternative therapy.. World J Dent.

[23] Rusdy H, Oes A, Siregar IB, Sitompul D (2018). The effectivity of ozone water application on tampon in post posterior tooth extraction in Department of Oral Surgery and Maxillofacial Faculty of Dentistry, University of North Sumatera (USU). J Dentomaxillofac Sci.

[24] Sen S, Sen S (2020). Ozone therapy a new vista in dentistry: integrated review. Med Gas Res.

[25] Chęciński M, Chęcińska K, Nowak Z, Sikora M, Chlubek D (2022). Treatment of mandibular hypomobility by injections into the temporomandibular joints: a systematic review of the substances used. J Clin Med.

